# Patient perceptions of primary care rapid respiratory microbiological point-of-care testing: a qualitative study

**DOI:** 10.1136/bmjopen-2025-099666

**Published:** 2025-06-25

**Authors:** Rebecca Clarke, Emily Brown, Alastair D Hay, Paul Mitchell, Matthew J Ridd, Liang Zhu, Lucy Yardley

**Affiliations:** 1 School of Psychological Science, University of Bristol, Bristol, UK; 2 Centre for Academic Primary Care, University of Bristol, Bristol, UK; 3 Health Economics and Health Policy, University of Bristol, Bristol, UK; 4 School of Psychology, University of Southampton, Southampton, UK

**Keywords:** Antibiotics, Diagnostic microbiology, QUALITATIVE RESEARCH, Primary Care

## Abstract

**Abstract:**

**Objectives:**

Rapid microbiological point-of-care tests (RM-POCTs) have the potential to reduce antimicrobial overuse for respiratory tract infections (RTIs). However, patient perspectives regarding RM-POCTs remain unclear. Therefore, this study aimed to explore patients’ and parents’ experiences using RM-POCTs for RTIs and their views on how RM-POCTs influence treatment decisions, symptom management and future consulting.

**Design:**

A qualitative study using in-depth, semistructured interviews. Data were analysed thematically, informed by a realist approach.

**Setting:**

Interviewees were recruited from a multicentre, individually randomised controlled efficacy trial evaluating the use of a multiplex RM-POCT for suspected RTIs in primary care.

**Participants:**

Purposive sample of primary care patients (n=21 adults, 9 parents) participating in the trial.

**Results:**

In general, participants viewed RM-POCTs favourably. Patients believed RM-POCTs reduced diagnostic uncertainty but emphasised that RM-POCTs should be used alongside clinical judgement. For some, additional information from RM-POCTs created positive outcome expectancies and reduced the perception that antibiotics were necessary. Others felt invalidated by RM-POCTs’ results or believed further support was necessary to understand when antibiotics were needed and how they could manage symptoms. While RM-POCTs may reduce reconsulting for the same illness, participants indicated future consulting behaviours would persist for self-limiting symptoms or health anxiety. Increased consulting may occur if patients perceive RM-POCTs to reduce pressure on primary care.

**Conclusion:**

RM-POCT offers the potential to improve self-efficacy beliefs and reduce reconsulting for the same illness. Effective clinician communication and patient education may be beneficial alongside RM-POCTs to minimise unintended outcomes and enhance patients’ ability to determine when primary care attendance is necessary in the future.

**Trial registration number:**

ISRCTN16039192

**STRENGTHS AND LIMITATIONS OF THIS STUDY**

- In-depth, one-on-one interviews facilitated a nuanced understanding of participants’ views and experiences.
- A realist-informed approach offers insight into how contexts can shape outcomes.
- A mostly white British sample from general practitioner (GP) practices in the least deprived areas may limit the generalisability of findings.

## Introduction

 The overuse and inappropriate prescription of antibiotics continue to fuel antimicrobial resistance (AMR).^1^ In 2019, an estimated 1.27 million deaths were directly attributed to AMR.^2^ If no action is taken, AMR is estimated to cause 10 million deaths annually by 2050.^1^ The UK government has outlined a 5-year national action plan to tackle AMR and declared improved targeting of antimicrobials a priority.^3^ An obvious area for intervention is the frequent prescription of antibiotics for respiratory tract infections (RTIs) in primary care,^4^ despite evidence indicating little clinical benefit for patients due to their commonly viral or self-limiting nature.^5 6^ This inappropriate prescribing not only exposes patients to unnecessary side effects but can reinforce patient treatment beliefs and promote health-seeking behaviours for similar illnesses in the future.^7 8^ Consequently, it is essential to implement strategies that support healthcare workers in safely reducing antibiotic prescriptions for RTIs.^2^

The 2020 Wellcome Trust AMR report^9^ and the UK Commission’s ‘Review on Antimicrobial Resistance’^10^ recommend the use of diagnostic tests that can distinguish viral from bacterial infection as a solution. Rapid microbiological point-of-care tests (RM-POCTs) have started to be trialled in primary care in the UK, with findings indicating clinician acceptability and the potential to reduce diagnostic uncertainty and improve antimicrobial use.^11 12^ Moreover, qualitative studies with clinicians suggest that RM-POCTs may be helpful for clinicians to address patients’ beliefs about antibiotic necessity.^11^

Nevertheless, patient perspectives towards RM-POCTs are largely unknown. In the wake of the COVID-19 pandemic, it is speculated that patients may be more accepting of respiratory tract samples being obtained.^13^ However, there have been concerns that diagnostic tests may have unintended consequences, such as increasing health-seeking behaviours and the demand for future testing.^13^ It is necessary to gain insight into patients’ perspectives on RM-POCTs to optimise implementation and reduce the risk of unintended consequences.^11 14^

### Research aims

This study aimed to explore patients’ and parents’ experiences using RM-POCTs for RTIs and their views on how RM-POCTs may influence treatment decisions, symptom management and their future consulting behaviours.

## Methodology

### Study setting

The qualitative study was embedded in a multicentre, individually randomised controlled efficacy trial to evaluate multiplex RM-POCTs for suspected RTIs in primary care.^15^ The multiplex RM-POCTs used in this trial were the BioFire RP2.1 plus reagent pouches with the BioFire FilmArray Torch 1.^16^ The test results indicate the presence or absence of 23 upper respiratory microbes—19 viruses: influenza A (no subtype detected, H1, H1-2009, H3), influenza B, adenovirus, Coronaviruses (HKU1, NL63, 229E, OC43, Mers-CoV, SARS-CoV-2), human metapneumovirus, human rhinovirus/enterovirus (not possible to distinguish due to genetic similarity), parainfluenza (types 1, 2, 3, 4) and respiratory syncytial virus (RSV) and four atypical bacteria: *Bordetella pertussis*, *Bordetella parapertussis*, *Chlamydia pneumoniae* and *Mycoplasma pneumonia*. Trial clinicians were provided with guidance on the typical presentation of illnesses caused by the microbes tested. However, final antibiotic prescribing decisions remained with the clinician.

Sixteen general practitioner (GP) practices in South West England were provided with a BioFire FilmArray Torch 1 and recruited patients between November 2022 and May 2024. Patients aged ≥12 months who presented to primary care with a suspected RTI where antibiotics might be necessary were eligible. ‘Appointment one’ comprised a standard clinical assessment, except that the treatment decision was deferred. Following consent, a trained member of staff took a nasal and throat swab from participants. Participants were then randomised to the intervention group (usual care plus RM-POCT result) or control group (usual care without RM-POCT result). Participants and their corresponding clinicians in the intervention group were provided with the RM-POCT result before ‘appointment two’, when participants were provided with treatment decisions. Clinicians were not provided with guidance on how to deliver RM-POCTs’ results or treatment decisions to patients and communication likely varied between clinicians. Control group participants received their treatment decision without a RM-POCT result.

### Sample and recruitment

After appointment two, participants who had also consented to be contacted about a qualitative interview were provided with more information. Adult patients (≥16 years of age) and parents and carers of child patients (<16 years of age) were purposively sampled to ensure variation in age, gender, ethnicity, intervention arm, treatment decisions and practice area deprivation. Henceforth, the use of parent refers to both parents and carers. Interested patients and parents contacted researchers to ask further questions and arrange an interview. The National Health Service (NHS) Research Ethics Committee approval was granted before recruitment (#22/NW/0294) commenced. Recruitment continued until data saturation was reached, and no new information was obtained from interviews that would add to the development of themes.

### Data collection

Individual interviews were conducted remotely between February 2023 and February 2024. A semistructured interview topic guide was developed from existing literature and study objectives (see online supplemental file 1). Topic guides supported discussion of participants’ experience using RM-POCTs for RTIs and their views on how RM-POCTs may influence treatment decisions, symptom management and future consulting. Written informed consent was collected before the interviews began. Interviews lasted an average of 32 min and were conducted by a researcher trained and experienced in qualitative health research (RC). Participants received a £10 voucher to thank them for their time.

### Data analysis

Interviews were audio-recorded, transcribed and anonymised. NVivo R1 software supported a thematic analysis informed by a critical realist approach.^17^ Realist approaches are particularly appropriate for evaluating complex interventions to understand why desired or adverse outcomes may occur.^18^ The identification of the settings or circumstances in which an intervention is implemented (context), the responses triggered by intervention resources (mechanism) and the outcomes from the interaction (outcome) allows for testable hypotheses to be developed.^18^

Analysis began by reading transcripts several times to become familiar with the data. Following this, RC inductively coded salient concepts in the transcripts line-by-line to stay close to the data. Team members reviewed a subset of transcripts and regularly met to resolve differences in interpretation. Following an iterative approach, codes were grouped to generate tentative themes that reflected patterns within and across the data. Theme generation was reviewed and refined by the team, and the final framework was applied to all transcripts.

Following the thematic analysis, RC and LY (behavioural scientist and professor) continued analysis using abductive reasoning. Abductive reasoning allows researchers to draw on both empirical data and theoretical insights from existing literature to hypothesise the underlying factors that can influence outcomes.^18^ In this case, abductive reasoning was drawn on to hypothesise the causal pathways that can lead to either positive or adverse outcomes from RM-POCTs’ implementation. To do this, RC and LY identified and interpreted the outcomes from interactions between contexts and mechanisms in which RM-POCTs were implemented. The analysis was repeated across the themes and enabled the development of context–mechanism–outcome (CMO) configurations to provide hypotheses of how positive or adverse outcomes from RM-POCTs use may have occurred. This two-step process aligns with previous qualitative research aiming to explore participant experiences and identify commonly occurring contexts, mechanisms and outcomes to develop or refine CMO configurations.^19 20^

### Patient and public involvement (PPI)

Patients and members of the public have been involved since the inception of the individually randomised controlled efficacy trial in which this qualitative study is embedded. The PPI group contributed to the trial design, including the mixed-methods investigation, and provided feedback on patient-facing documents. The Trial Management Group meetings also included PPI members, in which PPI members contributed to discussions on participant recruitment strategies and the qualitative findings.

## Results

Qualitative interviews were conducted with 30 participants recruited from 11 different GP practices (see table 1). An additional three trial participants provided consent to partake in a qualitative interview but became unresponsive. Data analysis generated four core themes: perceptions of RM-POCTs in consultations, desired outcomes from consultations and RM-POCTs, the wider context of implementation and patient factors. The CMO figurations created from the data and CMOs from the tables are referenced below in tables2  3.

**Table 1:** Patient and parent characteristics

Patients (n=21)	
Age (range)	29–77 years
Gender	Female: 11Male: 10
Intervention arm	Intervention: 13Control: 8
Treatment decision	Antibiotic prescription: 10No antibiotic prescription: 11
Practice area deprivation score*	1–3: 64–6: 37–10: 12
Chronic illness	Asthma: 2Diabetes: 2Heart failure: 1Bronchiectasis: 1
Ethnic group	White English, Welsh, Scottish, Northern Irish or British: 19Any other white background: 1Indian: 1
Parents (n=9)	
Child age (range)	1–15 years
Child gender	Female: 5Male: 4
Parent gender	Female: 8Male: 1
Intervention arm	Intervention: 5Control: 4
Treatment decision	Antibiotic prescription: 3No antibiotic prescription: 6
Practice area deprivation score	1–3: 14–6: 47–10: 4
Child chronic illness	Asthma: 3
Child ethnic group	White English, Welsh, Scottish, Northern Irish, or British: 6White Irish: 1Any other white background: 1White and Asian: 1

* The English Index of Multiple Deprivation Score,^35^ 1=most deprived, 10=least deprived.

**Table 2:** Positive outcomes from RM-POCTs on patient satisfaction, confidence in recovery without antibiotics and self-efficacy to self-manage illness presented in the CMO configurations

No	Context	Mechanism	Outcome
1	If patients are familiar with COVID-19 rapid tests and believe rapid tests have diagnostic value	…patients perceive the advantages of RM-POCTs outweigh the disadvantages (ie, discomfort, delayed treatment decisions)	…which increases patient acceptability of RM-POCTs
2	When patients believe rapid tests to be accurate	…patients will be confident in the use of RM-POCTs to support clinical diagnosis	…and confident in their ability to recover with or without antibiotics
5	When a patient trusts the clinician	…patients accept whether a RM-POCT is used to support patient management	…and patients are satisfied with the care received
7	In a context where patients have different levels of antibiotic knowledge	…the use of RM-POCTs enhanced patient recognition that antibiotics were not always necessary for respiratory infections	…and reduced patients’ expectations of receiving antibiotics in the future when attending primary care for respiratory symptoms. However, future consulting behaviours are uninfluenced due to ongoing patient uncertainty about which symptoms need antibiotics*
8	When patients are uncertain how to manage symptoms and they place value on patient-centred care, and therapeutic interactions alongside RM-POCTs’ use are perceived to be personalised with precise guidance	…patients acquire information on how to manage symptoms and feel empowered to self-manage	…As a result, patients are satisfied with care and confident in managing present/future symptoms
10	When patients have anxiety about their symptoms and/or low self-efficacy in their ability to recover without antibiotics	…the use of RM-POCTs enhances patients’ confidence in clinical diagnosis and treatment decisions	…As a result, patients’ confidence in their ability to self-manage without antibiotics increases, and re-presenting for the same illness and self-prescribing behaviours reduce. However, future consulting behaviours may not change if health anxiety arises.*
12	When patient consulting behaviours are influenced by concern about infecting vulnerable friends or family members	…patients value the additional information provided by RM-POCTs	…As a result, patients feel satisfied with care and avoid family members/friends until symptoms have resolved
15	When patients experience high levels of concern about antibiotic side effects or AMR	…patients perceive RM-POCTs to reduce unnecessary antibiotic consumption	…which increases patient satisfaction with care
16	When existing patient antibiotic necessity beliefs are shaped by prior positive experiences of receiving antibiotics	…patients appraise RM-POCTs and treatment decisions based on previous experiences and following the resolution of symptoms	…Patient recovery without antibiotics reduces antibiotic necessity beliefs and increases patients’ confidence in their ability to self-manage the future
18	When patients have existing vulnerabilities	…patients value RM-POCTs which indicate that antibiotics are necessary to reduce the risk of adverse outcomes that can happen from delayed access to antibiotics	…Thus, patients feel satisfied with care

* Unchanged consulting behaviours cannot be separated from CMO with other positive outcomes.

AMR, antimicrobial resistance; CMO, context–mechanism–outcome; RM-POCTs, rapid microbiological point-of-care tests.

**Table 3:** Adverse outcomes from RM-POCTs on patient satisfaction, confidence in recovery without antibiotics and self-efficacy to self-manage illness presented in the CMO configurations

No	Context	Mechanism	Outcome
3	When patients are uncertain about the accuracy of rapid tests	…patients will be distrustful of the use of RM-POCTs to support clinical diagnosis	…and will be less confident in their ability to recover
4	If a clinician is uncertain about the diagnosis, RM-POCTs’ results are inconclusive, and the clinician is perceived to not explore other causes of symptoms	…patients feel invalidated and believe there to be an overreliance on RM-POCTs	…As a result, patients have low satisfaction with the care received and confidence in the diagnosis
6	When a patient has low trust in a specific clinical role (eg, if the clinician is an allied health professional rather than a GP)	…the patient will have low confidence in the clinical interpretation of RM-POCTs’ results	…and low satisfaction with the care received and confidence in the diagnosis
9	If patients are uncertain how to manage symptoms and they place value on patient-centred care, but therapeutic interactions alongside RM-POCTs’ use are perceived to be generalised and unhelpful	…patients feel unsupported despite RM-POCTs' use	…and uncertain how to manage present/future symptoms
11	When patients experience difficulty getting GP appointments and perceive there to be pressure on the National Health Service (NHS)	…patient belief that RM-POCTs will save GP practices money and enhance clinic efficiency	…will create motivation for patients to consult earlier when experiencing symptoms and increase patient consulting behaviours
13	When patient self-efficacy beliefs and consulting behaviours are influenced by their social networks	…patients value the additional information provided by RM-POCTs	…and patients are motivated to share their diagnosis with their social network to promote consulting or self-management
14	When a parent anticipates their child’s school to request a doctor’s note to explain absence from school	…parents will continue to consult	…to provide evidence for non-attendance. Therefore, previous RM-POCTs’ use will reduce parents’ consulting behaviours
17	When patient antibiotic necessity beliefs are shaped by prior positive experiences of receiving antibiotics, existing patient vulnerabilities and/or illnesses highlighted in the media	…patients fear the consequences of RM-POCTs not supporting antibiotic use	…Consequently, despite RM-POCTs’ results indicating antibiotics are not necessary, patients have low confidence in their ability to recover without antibiotics and future consulting behaviours are maintained

CMO, context–mechanism–outcome; GP, General practitioner; RM-POCTs, rapid microbiological point-of-care tests.

### Perceptions of RM-POCTs in consultations

Most patients and parents viewed the testing experience positively. Performing swabs was described as *‘not that invasive’* (Patient 16) and they were generally considered quick and easy. The familiarity of swabs after the COVID-19 outbreak and previous use when living in other countries increased acceptance further (see CMO1, table 2). While not all participants viewed swabs favourably due to the discomfort experienced, the benefits of testing were considered to outweigh any negatives. Nevertheless, due to the *‘instantaneous natur*e’ (Patient 3) of the society and familiarity with rapid COVID-19 tests, some patients were frustrated to have to wait for results:

I think people are used to that speed in terms of like diagnosis… If the doctor would be able to diagnose you anyway, you could question the kind of need for it. (Patient 3)

In general, patients believed that RM-POCTs’ results could provide an objective answer as to whether antibiotics would be effective and, therefore, could optimise treatment approaches. This additional diagnostic information was perceived to be particularly important for children: *‘you’re trying to interpret someone else’s symptoms and that’s a challenge’* (Parent 9). The belief that RM-POCTs would have been robustly tested further increased the confidence of one participant (see CMO2, table 2). While participants showed an incomplete understanding of the limitations of RM-POCTs, a few participants recognised that rapid test results may be incorrect (see CMO3, table 3):

We’ve seen with COVID, there’s any number of ways that you could fail a test. So, you might question that more so than the actual doctor who’s looking in your throat or listening to your chest. (Patient 3)

Two participants speculated that younger patients might accept and trust RM-POCTs more than older generations.

Participants recognised that clinical judgement was still necessary alongside RM-POCTs and believed RM-POCTs should be used as a second opinion. Clinical examinations were considered essential to identify illnesses and other concerns that the RM-POCTs couldn’t detect:

They can spot any number of illnesses or maybe signs of social issues like sexual abuse and things like that, so… I wouldn’t necessarily say you want to take off the face-to-face entirely. (Patient 3)

In particular, parents who expressed heightened anxiety around their children’s well-being stressed that clinical examinations were crucial to ensure nothing was missed. However, a few patients worried that use of RM-POCT may reduce clinical judgement, prevent thorough consideration of symptoms or the diagnosis of coexisting illnesses (see CMO4, table 3):

I really think that in my case, where there was no conclusion at all *(from the RM-POCT*) over whether it was bacterial or viral, then the care should not have stopped. (Patient 5)

Participants’ perceptions about their clinician may also influence their view of RM-POCTs. For many participants, RM-POCTs’ acceptance stemmed from trust in their clinician (see CMO5, table 2). However, some participants recognised that RM-POCTs could be interpreted differently (see CMO6, table 3). While most participants were unconcerned about who reviewed RM-POCTs’ results, confidence in RM-POCTs’ use and diagnosis varied depending on participants’ perceptions of clinical roles:

I want it to be a GP, rather than going to the pharmacist *[…]* what doctors do is completely different, wildly different to what pharmacists do which is literally symptoms and associated drugs, and drugs and interactions with other drugs. (Parent 5).

### Desired outcomes from consultations and RM-POCTs

Patients desired more information about antibiotic effectivity alongside RM-POCTs, believing that educational information was also necessary to promote self-management and reduce perceptions of antibiotic necessity:

That will help them to understand that things can be solved… in a natural way, you know, with time. (Patient 11)

Nevertheless, increasing patients’ understanding of antibiotics may not be enough to enhance patients’ beliefs about their ability to self-manage in the future. Instead, many participants reported that ongoing uncertainty about which symptoms need antibiotics would continue to drive future consulting: *‘I don’t think you can tell from the symptoms. That’s a doctor thing, not a personal thing’* (Patient 9). Participants reported that severe, ongoing or unfamiliar symptoms would prompt future consultation, preferring to leave antibiotic prescribing versus self-management decisions to a clinician (see CMO7, table 2). One parent suggested that supporting patients’ ability to make informed decisions about when it was necessary to consult, such as with health apps, would reduce consulting behaviours.

Participants also sought holistic guidance on managing symptoms following RM-POCTs’ use: *‘I’m going in and I’m thinking, “I want to come out with a plan”’* (Parent 5). The provision of *‘precise advice’* (Parent 7) to ease symptoms that also accounted for personal factors (eg, coexisting illnesses) and a timeline of when to reconsult following RM-POCTs’ use increased parents’ and patients’ perceptions of their ability to self-manage and, as a result, may reduce future consulting for similar symptoms (see CMO8, table 2):

But more importantly, it’s given me as a parent the information and the confidence to do that again, and I won’t need to see a doctor. (Parent 5)

In contrast, perceptions of generalised interactions without guidance on how to self-manage symptoms increased frustration in participants who were not prescribed antibiotics in both the intervention and control group, suggesting that RM-POCT use alone will not always increase patients’ confidence to self-manage (see CMO9, table 3):

I did really feel like I was just left hanging to sort of like, try and decipher myself what I should be doing. (Patient 5)

While participants valued RM-POCTs, patient-centred interactions in which clinicians listened to *‘the person living in the body’* (Parent 5) were still viewed as essential. Healthcare, well-being and treatment should be a joint decision in which a patient or parent can make an informed choice. Rather than enabling personalised care, a few participants worried that RM-POCTs may reduce clinician–patient interaction and collaboration on treatment decisions.

Does that mean that a 10-minute appointment gets forced as a five-minute appointment and then you lose that interaction ability? (Patient 15)

Participants highlighted that their consulting behaviours could be influenced by worry about symptoms and their ability to recover without antibiotics. Results from RM-POCTs that suggested antibiotics were unnecessary provided peace of mind and enhanced beliefs that patients could recover without treatment, elucidating the potential for RM-POCTs to reduce reconsulting and self-prescribing behaviours in some participants (see CMO10, table 2):

Otherwise you come away and you think, okay, I’ll give it another couple of days and maybe I’ll see another doctor and they’ll tell me something different. It just takes away all of that doubt. (Patient 16)

For example, had I not gone the way of antibiotics then, and being convinced myself that I needed it, I potentially may have looked at trying to find some old *(antibiotics*) in the cupboard and used what was prescribed. (Patient 3)

However, some participants believed further verbal reassurance from clinicians was still necessary following RM-POCTs. Addressing any existing concerns was particularly important if RM-POCTs created more uncertainty about the cause of (coexisting) symptoms:

The experience I had seemed a bit confusing *[…]* It was sort of, we can’t do anything while it looks like you’ve got Covid. (Patient 4)

Moreover, previous use of RM-POCTs may not influence future consulting if motivation to consult stems from health concerns and hope for emotional support from clinicians:

I don’t think it would change anything personally, because you’d still… like my motivation for going would be to get treatment or I need to get some reassurance that I’m actually, you know, fixing myself, if you like. (Patient 10)

### The wider context of implementation

Participants considered the context in which RM-POCTs were implemented. While it was acknowledged that the provision of RM-POCTs would cost money, participants thought they would help GP practices and the NHS financially through reduced unnecessary antibiotic prescriptions and consultations:

I know some of them will incur additional expense, but at the saving of money being wasted elsewhere. (Patient 1)

Despite beliefs that clinical assessment alongside RM-POCTs was necessary, some patients raised that they would be willing to pay for RM-POCTs at a pharmacy to get a diagnosis and reduce pressure on the NHS, highlighting that the commercialisation of tests could be accepted: *‘you wouldn’t really potentially need to go and see a doctor’* (Patient 20). However, one patient expressed frustration at healthcare services moving away from GP practices.

Additionally, clinic efficiency was thought to improve if RM-POCTs were used. Patients expressed a sense of hesitance and guilt in using GP practices due to pressure on NHS services. In contrast to suggestions that clinical examination and patient-centred care were still essential with RM-POCTs’ use, some participants believed that RM-POCTs could reduce the strain on the NHS if they were used as a triage tool to *‘weed out’* (Patient 3) people who do not need appointments. Thus, patients may feel less of a *‘nuisance’* (Patient 17) and be more likely to attend their GP practice earlier if they perceive RM-POCTs to enhance clinic efficiency and ease pressure on the NHS (see CMO11, table 3):

I'd rather obviously keep going to pick one of those tests up and then do the test, rather than waste the doctor’s time for something that you've literally just got to sort through. (Patient 20)

I guess maybe that would change me in terms of actually I might go to the doctors a bit sooner as opposed to… to kind of toughing it out or not. (Patient 21)

Participants raised a sense of socially responsible consulting behaviours. Some patients believed they needed to resolve symptoms so as not to transfer their illness to their social network. These participants felt RM-POCTs’ results could help prevent contagious illnesses from spreading to vulnerable friends or family (see CMO12, table 2). While it was acknowledged that it was *‘up to someone else’* (Patient 10), sharing RM-POCTs’ results was perceived to promote socially responsible consulting and self-management behaviours (see CMO13, table 3). It was considered that sharing viral results with friends experiencing similar symptoms could discourage unnecessary consulting: *‘I’m going to kind of like say, oh, “you don’t need to do this because I’ve gone…”’* (Patient 10). Two parents believed it would be *‘respectful’* (Parent 2) to share bacterial diagnoses with their child’s nursery and school:

It means that, you know, if the other kids start presenting symptoms, they’ve got a bit more agency of knowing what it is. (Parent 2)

Moreover, parents considered the practical implications for their children’s school as consulting and RM-POCTs’ results could provide evidence for non-attendance (see CMO14, table 3):

I always have to take my son when he’s unwell, because then he doesn’t go to school. So, I need to have a back-up if you know what I mean. (Parent 1).

### Patient factors

Participants’ understanding of antibiotics varied. Several participants held conflicting views as they desired antibiotics for quick recovery, while also being worried about consuming too many antibiotics. Thus, RM-POCTs were valued as a way to reduce unnecessary antibiotic consumption by those concerned about AMR or the short-term side effects of antibiotics (see CMO15, table 2).

Antibiotics wipe us out. You know, that leaves us with a different debt to pay *[…]* I want to make sure that I've intervened in every way possible. (Parent 5)

Participants’ antibiotic necessity beliefs stemmed from prior illness experiences. Participants believed that antibiotics were necessary when consulting with symptoms similar to those on previous occasions when antibiotics were prescribed. Recovery without antibiotics on this occasion altered this belief in patients in both the intervention and control group (see CMO16, table 2):

This experience has shown me that I didn’t have antibiotics, I felt really ropey, but I got over it without antibiotics. So, it’s obviously not the be all and end all, is it? Looking after yourself, rest, hydration, that obviously can do the trick as well. (Patient 16)

However, the risk of long-term complications or symptoms worsening remained a concern for some participants. These intervention and control group participants reported that they still desired precautionary antibiotics in the future to alleviate symptoms or prevent long-term complications (see CMO17, table 3). Moreover, media coverage of illnesses, such as Strep A and long covid, left individuals *‘scared’* (Parent 2) about severe complications, suggesting ongoing uncertainty about antibiotic effectiveness and maintained antibiotic necessity beliefs for both viral and bacterial infections.

For other patients, illness experiences and RM-POCTs were assessed in the context of perceived illness vulnerability. Factors such as age, perceived immune system weakness, chronic health conditions and virus outbreaks in school increased concern about symptoms. The use of RM-POCTs was perceived to reduce the heightened risk of adverse outcomes with vulnerabilities when antibiotics are delayed (CMO18, table 3):

I've got an autoimmune condition, so sometimes when I get illnesses, it makes it a lot worse *[…]* So, for me, it’s really helpful to find out straight away that I can have the tablets, rather than having to just potentially deal with it getting worse. (Patient 20)

Some vulnerable patients and parents in the intervention group who received antibiotics feared the consequences of not receiving antibiotics again when ill (see CMO17, table 3):

I’m a bit scared because it’s your lungs, isn’t it, and I’m an old woman *[…]* I think I might get pneumonia and die. (Patient 18)

Consequently, it was considered safer to continue to consult rather than self-manage.

## Discussion

### Summary

In general, participants viewed RM-POCTs favourably. Patients believed RM-POCTs were a valuable tool for clinicians to reduce diagnostic uncertainty but emphasised that RM-POCTs should be used alongside clinical judgement in patient-centred consultations. For some patients, additional information from RM-POCTs alleviated health anxiety and enhanced perceptions that they could recover without antibiotics. Others felt invalidated by RM-POCTs’ results or believed further support was necessary to help patients understand when antibiotics are necessary and how they can manage symptoms. Most patients expected to continue to consult as usual in the future when they perceived symptoms to be severe or they wanted reassurance. A few patients suspected that consulting behaviours may increase if RM-POCTs were perceived to reduce the burden on primary care. Further quantitative analysis and the qualitative findings exploring clinicians’ experiences with RM-POCTs in the RAPID-TEST trial will be reported separately.^15 21^

### Strengths and limitations

To the authors’ knowledge, this is the first qualitative study exploring patients’ and parents’ views and experiences with RM-POCTs. Participants were purposively sampled from 11 GP practices participating in the RM-POCTs’ trial to ensure that different participant characteristics and experiences were included. This variety helped capture varying satisfaction levels, demonstrating that positive and adverse outcomes can occur with RM-POCTs.

Nevertheless, the representativeness of the participants was limited by a mostly white British sample from GP practices in the least deprived areas. This sample reflects the nature of this efficacy trial, whereby 95% of participants were white British and may have implications for the generalisability of the findings across the UK. It is also possible that patients’ perspectives of RM-POCTs were influenced by the delivery of intervention content and clinicians’ views of the tests.^21 22^ However, this unscripted approach to intervention delivery reflects the real-world setting in which RM-POCTs would be implemented.

Additionally, while critical realist principles informed the consideration of the context and mechanisms that may generate outcomes, this study focused only on the immediate context of the clinician–patient dyad, primary care and broader influences raised by participants; we are aware that there are wider contexts outside the scope of this study.

### Comparison with existing literature

Consistent with previous research on C reactive protein point-of-care testing (CRP-POCT),^23 24^ we found that some participants reported increased confidence in prescribing decisions when RM-POCTs were used. However, when results did not align with participants' illness experience and antibiotic expectations, RM-POCTs did not always persuade patients that antibiotics were unnecessary.^23 24^ Our study extends this understanding by suggesting that patients’ doubts about RM-POCTs’ interpretation may stem from their low confidence in allied healthcare professionals (AHPs) expertise. This finding contrasts with a study by Czarniak *et al,* which found that pharmacists perceived CRP-POCTs to enhance their professional credibility^25^ and may have implications for the growing role of AHPs supporting healthcare in the community. Our findings suggest that effective clinician communication skills, alongside RM-POCTs, are essential to address patient knowledge about antibiotics, the limitations of RM-POCTs and misconceptions about what negative/inconclusive RM-POCTs mean, and to thoroughly explain prescribing decisions.^26 27^ Additional communication strategies should also address patient anxiety when antibiotics are not prescribed, as evidence suggests that education alone may not successfully modify patient beliefs and behaviours.^28 29^ As previous findings highlight that communication skills training has a more sustainable impact on reducing antibiotic prescribing for RTIs than CRP-POCT,^30^ the cost-effectiveness of antimicrobial strategies must be considered.

This present study demonstrates that RM-POCTs may enhance some patients' self-efficacy and perceptions of their ability to recover without antibiotics. In turn, this may reduce reconsulting for the same illness. However, in line with existing research using CRP-POCT, many participants indicated that their future consulting behaviours would persist for self-limiting symptoms.^23 27^ This finding suggests that patients will also require additional support to determine when future consulting is necessary if RM-POCTs are implemented as a way to reduce consulting behaviours.^31^ Previous studies have demonstrated that providing support on managing RTI symptoms and guidance on when to attend primary care can improve patient satisfaction, reduce emotional drivers of consulting and enhance self-efficacy to self-manage symptoms.^27 32^ These complementary educational approaches may be particularly beneficial for individuals who feel unsupported when antibiotics are not prescribed following RM-POCTs’ use, patients with vulnerabilities who have lower self-efficacy beliefs or those who may associate consulting for RM-POCTs with future RTI management.^32 33^

### Implications for research and practice

Our findings highlight how RM-POCTs offer the potential to create positive outcome expectations and reduce perceptions that antibiotics are necessary to recover. Notably, there are many similarities in patient experience using RM-POCTs and CRP-POCT, suggesting that RM-POCTs offer a viable alternative to CRP-POCT, to which uptake has remained low in primary care.^13^ Nevertheless, positive outcomes do not occur with all patients. Additional strategies, such as effective communication and patient education, should be considered alongside RM-POCTs to support patient satisfaction with care, address patient medication beliefs and self-efficacy, and enhance patients’ ability to determine when primary care attendance is necessary. The context–mechanism–outcome configurations identified in this study offer insight into the contexts, whereby further strategies may be the most useful. Future research should identify which strategies are the most effective alongside CRP-POCT while considering the resource pressures faced in primary care to enhance sustainability.^30 34^ Further research should also consider exploring whether RM-POCTs’ experiences vary across ethnic groups and with patients from underserved areas, so that tests can be optimised across the UK.

## Supplementary material

10.1136/bmjopen-2025-099666 online supplemental file 1

## Data Availability

At present, data is available on request from the authors. However, the data will be made open access at a later date along with other data from the trial.
