# Supplementary material for: Patient perceptions of primary care rapid respiratory microbiological point-of-care testing: a qualitative study
Source: BMJ Open. 2025 Jun 25;15(6):e099666. doi: 10.1136/bmjopen-2025-099666 (PMC12198824; doi:10.1136/bmjopen-2025-099666)
Supplement: online supplemental file 1 [file bmjopen-15-6-s001.docx]

**Supplementary File 1: Patient/Parent Interview Topic guide**

Intervention participant interview topic guide

1. Can you tell me a bit about your reason for visiting the GP (with your child) on this occasion?
2. What were you told about the new POCT^RM^ for respiratory infections?
   1. What were your initial thoughts (and your child’s initial thoughts) about this test? Why?
3. Did you (and your child) choose to have the POCT^RM^?
   1. Why did you make that choice?
   2. Did you or your child have any concerns or worries about taking the test?
4. Can you describe how the test was performed and how you/ your child found that experience?
   1. Who performed the POCT^RM^? How did you/ your child feel about that?
   2. How was the POCT^RM^ performed?
   3. How did you/ your child find that experience?
   4. What about this process went well?
   5. How could this process be improved?
5. What happened after the test was performed?
6. Can you tell me a bit about what information the POCT^RM^ provided?
   1. How useful is it for the patient/ parent to receive that information?
   2. How useful is it for the clinician to receive that information?
   3. How do you feel about different clinicians looking at the test results and deciding treatment outcomes? E.g. Should it be a doctor looking at the test result?
7. How did the POCT^RM^ result influence the treatment of your symptoms/ your child’s symptoms?
   1. How do you feel about the test influencing that decision?
   2. Did you/ your child have any concerns?
8. Did the POCT change how you normally communicate with your doctor about your symptoms and treatment? If so, how?
9. After this experience, what would you do if you/ your child had similar symptoms in the future?
10. Is there anything else you would like to add?

Control participant interview topic guide

1. Can you tell me a bit about your reason for visiting the GP (with your child) on this occasion?
2. What were you told about the POCT^RM^ for respiratory infections?
   1. What were your (and your child’s) initial thoughts about this test? Why?
3. Would you choose (for your child) to have POCT^RM^ in the future?
   1. Why?
   2. Would you/ your child have any concerns or worries about taking the test?
4. How useful would it be for the patient/ parent to receive POCT^RM^ results? Why?
5. How useful would it be for the clinician to receive POCT^RM^ results? Why?
6. How do you feel about different clinicians looking at the POCT^RM^ results and deciding treatment outcomes? E.g. Should it be a doctor looking at the test result?
7. Would a POCT^RM^ change how you normally communicate with your doctor about your symptoms and treatment / your child’s symptoms and treatment? If so, how?
   1. Why?
8. How did you manage your symptoms/ your child’s symptoms on this occasion?
   1. Do you think you would have managed the symptoms differently if you had the POCT^RM^? How so?
   2. Did you (or your child) have any concerns about managing your symptoms without a POCT^RM^?
9. After this experience, what would you do if you/ your child had similar symptoms in the future?
10. Is there anything else you would like to add?
