# Supplementary material for: Patient perceptions of primary care rapid respiratory microbiological point-of-care testing: a qualitative study
Source: BMJ Open. 2025 Jun 25;15(6):e099666. doi: 10.1136/bmjopen-2025-099666 (PMC12198824; doi:10.1136/bmjopen-2025-099666)
Supplement: Supplementary file 2 [file bmjopen-2025-099666.reviewer_comments.pdf]

PEER REVIEW HISTORY

BMJ Open publishes all reviews undertaken for accepted manuscripts. Reviewers are asked to complete a checklist review form and are provided with free text boxes to elaborate on their assessment. These free text comments are reproduced below.

ARTICLE DETAILS

Title (Provisional)

Patient perceptions of primary care rapid respiratory microbiological point-of-care-testing: a qualitative study

Authors

Clarke, Rebecca; Brown, Emily; Hay, Alastair D; Mitchell , Paul; Ridd, Matthew J; Zhu, Liang; Yardley, Lucy

---

VERSION 1 - REVIEW

**Reviewer**	**1**
**Name**	**Bakon, Sophia**
**Affiliation**	**National Institutes of Health, Institute for Medical Research**
**Date**	**05-Mar-2025**
**COI**	**None**

---

1. Is the research question or study objective clearly defined?- Please write in a point objective so that clearer to follow especially in the Introduction section (aim)

2. If statistics are used are they appropriate and described fully? Not clearly described in the data analysis. Sample size should be mentioned

3. Are the study limitations discussed adequately? such as drop out and number of patients recruit for the study, sample size issues

---

**Reviewer**	**2**
**Name**	**Gant, Vanya**
**Affiliation**	**University College Hospital, Microbiology**
**Date**	**22-Apr-2025**
**COI**	**None**

This is a relatively small but very timely and interesting study in this seriously underappreciated and hugely important field of (in the broadest sense) technology adoption

in healthcare. Unlike large pathology laboratories, successful deployment of POCT requires both local knowledge of the tests' capabilities – but, equally importantly, the interactions that flow from its results between the POCT itself, the treating clinician and the patient. It is rapidly becoming clear that technology itself may be at best ineffective and at worse dangerous if not deployed and interpreted correctly.

This study asks about what patients think about the test and its results; and the answers given are both enlightening and worrying. The "Results" here often relate to statements made by patients – many very thoughtful and considered - and I am a great believer in the Power of the Narrative in research situations where the concept of the RCT adds little value to the message.

The authors also structured these answers according to context-mechanism-outcome configurations, which were clearly explained.

The bottom lines for this "real life" study are salutary. And like real life, these suggest both considerable variation in how patients view and trust such tests, and why the simplistic view that a high performance diagnostic must automatically be a Good Thing is incorrect.

The discussion is thoughtful and (in my opinion) balanced – concluding that much work beyond technology itself needs to be done, for the correct deployment of a Good Test to lead to Better Care.

I was also very interested in the diversity of opinion as concerns the "need" for a doctor in this system. The UK cradle-to-grave NHS mantra brings with it the potential for over-reliance on its medical systems. The diversity of opinion on pharmacist-delivered testing was particularly interesting. Many such POC tests are already deployed in French pharmacies (as one example), and French people would not expect to bother a doctor for URT symptoms and follow the pharmacists' advice (including in some cases referral to a doctor for an antibiotic!).

I suggest that the authors might also provide a short section on what the test detects (I did find what this was, buried in a paper referencing the larger study). I say this because there are many such tests out there now – and most, with far more restricted pathogen panels. Readers will want to know without looking up the original paper. I presume that there was no "structured response" provided to the GPs as concerns how they presented the results to patients. If that is the case, it should be stated. One of the papers' unresolved issues (because it was not designed to do this) relates to possible variation in the GP's relationship and approach to the patients – they might, for example, unknowingly influence patient responses and decisions because of their own views of the POCT. Another study has demonstrated that a considerable proportion of doctors either don't believe, or don't trust, such new technologies1. There is potential for bias here –this is not the focus of this particular paper, but maybe should be mentioned, as there is likely as much variation in doctors' attitudes as these authors found in their patients.

In summary, this paper is refreshingly “different” and adds much to the issue of New technology implementation a very important and expensive field of medicine in volume terms and AMR generation.

With some minor additions, it should not only be published, but also give pause for thought for those in Healthcare who think that technology can lead. It prompts the concept of “technology +” – this representing the whole package of Test, clinical interpretation and optimising effective messaging to patients. If Doctors and patients were not human, we wouldn’t need such important research to continue.

1. 10.1093/jac/dkad355

VERSION 1 - AUTHOR RESPONSE

Reviewer: 1

1. Is the research question or study objective clearly defined?- Please write in a point objective so that clearer to follow especially in the Introduction section (aim)

   A ‘Research Aims’ sub-heading has now been included. We hope this can help enhance clarity.

2. If statistics are used are they appropriate and described fully? Not clearly described in the data analysis. Sample size should be mentioned

   This manuscript reports the findings from a qualitative study embedded within a multi-centre, individually randomised controlled efficacy trial. The qualitative methodology has been described in detail on Pages 2-4, including a reference to recruitment continuing until data saturation is reached. The sample size has been reported in the abstract on Page 1 (“n=21 adults, 9 parents”) and in the results on Page 4 (“Qualitative interviews were conducted with 30 participants recruited from 11 different GP practices”), in line with usual practice for qualitative research. In the Discussion on Page 11, we specify that statistical analysis from the trial will be reported separately.

3. Are the study limitations discussed adequately? such as drop out and number of patients recruit for the study, sample size issues

   Qualitative methodology prioritises depth of understanding over large sample sizes, with appropriate sample sizes determined by the richness of data and comprehensive insights into the phenomenon being studied (Vasileiou et al., 2018; Morse et al., 2000). The sample size, data collection and analysis sufficiently highlighted the multifaceted nature of how contextual factors can influence participants' perceptions and experiences of point-of-care testing. These nuanced insights directly addressed the research aims of this qualitative research. We recognise there may be sampling issues (i.e. the diversity of participants) and this has been reported in the limitations section:

"Nevertheless, the representativeness of the participants was limited by a mostly White British sample from GP practices in the least deprived areas. This sample reflects the nature of this efficacy trial whereby 95% of participants were White British and may have implications for the generalisability of the findings across the UK."

Three additional trial participants initially provided consent to partake in a qualitative interview but did not attend the scheduled interview and became unresponsive despite follow-up attempts. The following statement has been added to Page 4 to address this:

"An additional three trial participants provided consent to partake in a qualitative interview but became unresponsive."

Reviewer: 2

1. I suggest that the authors might also provide a short section on what the test detects (I did find what this was, buried in a paper referencing the larger study). I say this because there are many such tests out there now – and most, with far more restricted pathogen panels. Readers will want to know without looking up the original paper.

   Thank you for this suggestion. The following section has been added to the Methodology on Pages 2-3:

   "The rapid microbiological multiplex point-of-care tests used in this trial were the BioFire® RP2.1 plus reagent pouches with the BioFire® FilmArray® Torch[16]. The test results indicate presence or absence of 23 upper respiratory microbes: 19 viruses (Influenza A (no subtype detected, H1, H1-2009, H3), Influenza B, Adenovirus, Coronaviruses (HKU1, NL63, 229E, OC43, Mers-CoV, SARS-CoV-2), Human Metapneumovirus, Human Rhinovirus/ Enterovirus (not possible to distinguish due to genetic similarity), Parainfluenza (types 1, 2, 3, 4) and RSV and four atypical bacteria: Bordetella pertussis, Bordetella parapertussis, Chlamydia pneumoniae and Mycoplasma pneumonia. *Trial clinicians were provided with guidance on the typical presentation of illnesses caused by the microbes tested. However, final antibiotic prescribing decisions remained with the clinician."*

2. I presume that there was no "structured response" provided to the GPs as concerns how they presented the results to patients. If that is the case, it should be stated.

   While clinicians were provided with guidance on the typical presentation of illnesses caused by the microbes tested, clinicians were not provided with scripts or guidance on how to deliver results or treatment decisions to patients. The following statement has been added to Page 3:

   "Clinicians were not provided with guidance on how to deliver RM-POCT results or treatment decisions to patients and communication likely varied between clinicians."

3. One of the papers' unresolved issues (because it was not designed to do this) relates to possible variation in the GP's relationship and approach to the patients – they might, for example, unknowingly influence patient responses and decisions because of their own views of the POCT. Another study has demonstrated that a considerable proportion of doctors

either don't believe, or don't trust, such new technologies[1]. There is potential for bias here – this is not the focus of this particular paper, but maybe should be mentioned, as there is likely as much variation in doctors' attitudes as these authors found in their patients.

> *Thank you for this insight. The following statement has been added to the strengths and limitations section on Page 11:*
>
> *"It is also possible that patients' perspectives of RM-POCT were influenced by the delivery of intervention content and clinicians' views of the tests*[**Error! Reference source not found.,Error! Reference source not found.**]*. However, this unscripted approach to intervention delivery reflects the real-world setting in which RM-POCT would be implemented."*
